# Paeonol Ameliorates Glucose and Lipid Metabolism in Experimental Diabetes by Activating Akt

**DOI:** 10.3389/fphar.2019.00261

**Published:** 2019-03-19

**Authors:** Futian Xu, Haiming Xiao, Renbin Liu, Yan Yang, Meng Zhang, Lihao Chen, Zhiquan Chen, Peiqing Liu, Heqing Huang

**Affiliations:** ^1^Laboratory of Pharmacology and Toxicology, School of Pharmaceutical Science, Sun Yat-sen University, Guangzhou, China; ^2^Department of Endocrinology, The First Affiliated Hospital of Guangzhou University of Traditional Chinese Medicine, Guangzhou, China; ^3^Department of Traditional Chinese Medicine, Renmin Hospital, Hubei University of Medicine, Shiyan, China

**Keywords:** hyperglycemia, dislipidemia, paeonol, Akt, GCK and LDLR

## Abstract

Our previous study proved that paeonol (Pae) could lower blood glucose levels of diabetic mice. There are also a few reports of its potential use for diabetes treatment. However, the role of Pae in regulating glucose and lipid metabolism in diabetes remains largely unknown. Considering the critical role of serine/threonine kinase B (Akt) in glucose and lipid metabolism, we explored whether Pae could improve glucose and lipid metabolism disorders via Akt. Here, we found that Pae attenuated fasting blood glucose, glycosylated serum protein, serum cholesterol and triglyceride (TG), hepatic glycogen, cholesterol and TG in diabetic mice. Moreover, Pae enhanced glucokinase (GCK) and low-density lipoprotein receptor (LDLR) protein expressions, and increased the phosphorylation of Akt. In insulin-resistant HepG2 cells, Pae increased glucose uptake and decreased lipid accumulation. What’s more, Pae elevated LDLR and GCK expressions as well as Akt phosphorylation, which was consistent with the *in vivo* results. Knockdown and inhibition experiments of Akt revealed that Pae regulated LDLR and GCK expressions through activation of Akt. Finally, molecular docking assay indicated the steady hydrogen bond was formed between Pae and Akt2. Experiments above suggested that Pae ameliorated glucose and lipid metabolism disorders and the underlying mechanism was closely related to the activation of Akt.

## Introduction

Type 2 diabetes mellitus (T2DM) is increasing at an alarming rate and has become a global challenge. T2DM is a metabolic disorder characterized by insulin resistance (IR) and pancreatic β-cells dysfunction as a consequence of unsettled hyperglycemia (Butler et al., 2003). Glucose and lipid metabolism disorders are the main characteristics of insulin-resistant type2 diabetes mellitus (Kalsi et al., 2015). These disorders are also the basic pathology in diabetic microvascular complication (Stratton et al., 2000). Stable glucose-lowering and lipid-lowering therapies effectively retard the progression of chronic diabetic microvascular complications (Holman et al., 2008; Aschner and Ruiz, 2012). In T2DM, IR worsens the glucose and lipid metabolism disorders. Thus, strategies through the improvement of insulin signaling are of great importance to retain the homeostasis of glucose and lipid metabolism.

Serine/threonine kinase B plays a key role in insulin signal pathway, and regulate glucose and lipid metabolism in various aspects. Activated Akt inhibits GSK-3β kinase activity (Lee and Kim, 2007) and increases GCK activity by phosphorylation, leading to the promotion of glycogenesis and glycolysis (Tappy et al., 1997; Lin and Accili, 2011). Moreover, Akt influences *de novo* lipogenesis by regulating sterol regulatory element-binding transcription factor (SREBP-1c), and subsequently increasing LDLR protein expression (Horton et al., 2002; Guo et al., 2011). Therefore, it is of therapeutic significance to target Akt to improve insulin sensitivity for glucose and lipid metabolism disorders treatment (Maiese et al., 2007).

Paeonol (Pae, 2′-hydroxy-4′-methoxyacetophenone), a simple phenolic compound, is the main active component extracted from Moutan Cortex and *Cynanchum paniculatum.* Others have showed its biological and pharmacological activities regarding anti-inflammation (Chou, 2003), anti-oxidation (Chen et al., 2012) and anti-atherosclerosis (Yang et al., 2010), etc. Besides, there are a few reports of its potential use for the treatment of diabetes (Lau et al., 2007; Juan et al., 2010). Our preliminary study suggests that Pae could ameliorate diabetic nephropathy through its anti-oxidative effects via activation of silent information regulator 2-related protein 1/ nuclear factor E2-related factor 2 (Sirt1/Nrf2) (Zhang et al., 2018). However, its exact role in regulating glucose and lipid metabolism in experimental diabetic models and the underlying mechanisms remain unclear. Further consideration is taken into the vital role of Akt in glucose and lipid metabolism, and great attentions are focused on whether Pae works by affecting Akt.

Based on the above background, we used both *in vivo* and *in vitro* models in this study, i.e., diabetic mice models induced by low-dose of streptozocin combined with high-fat diet and insulin-resistant human hepatoma HepG2 cells induced by PA, to observe the effects of Pae on glucose and lipid metabolism as well as to explore its mechanism through Akt pathway. To further elucidate its interaction with Akt, Pae was docked into the active pocket of Akt2 to analyze the specific binding motif between them. Altogether, our results showed that Pae improve glucose and lipid metabolism through activation of Akt.

## Materials and Methods

### Reagents and Antibodies

Fetal bovine serum was purchased from Bovogen (Melbourne, VIC AU). Pae (purity > 99.0%, HPLC) in cell experiment and steptozocin (STZ) in animal study were from Sigma–Aldrich Corporation (St. Louis, MO, United States). Pae used in animal trial was from Zelang (purity > 98.0%, HPLC; Nanjing, China). Met Hydrochloride Tablets used in animal experiments were purchased from Bristol-Myers Squibb Company (Shanghai, China). MK-2206 was from Selleck Chemicals (Houston, TX, United States). SC79 was purchased from MedChemExpress (Monmouth Junction, NJ, United States). Insulin was from Beyotime Biotechnology (Haimen, China). 2NBDG was from Invitrogen (Carlsbad, CA, United States).

Primary antibodis against p-Akt (catalog: 66444-1-Ig) and Akt (catalog: 10176-2-AP) were from Proteintech Group (Wuhan, China). Antibodies against p-GSK3β (catalog: #9336) and GSK3β (catalog: #9315) were from Cell Signaling Technology (Boston, MA, United States). Antibody against GCK (catalog: A6293) was from ABclonal Technology (Boston, MA, United States). LDLR (catalog: ab30532) was from Abcam (Cambridge, MA, United States). HRP-conjugated secondary antibodies were acquired from Promega Corporation (Madison, WI, United States).

### Animal Experiment

Animal experiments were carried out as previously described (Yang et al., 2018). Male C57/BL6 mice aged 6 weeks weighing (20 ± 2 g) were supplied by the Experimental Animal Center of Sun Yat-sen University (Guangzhou, China). All experiments were approved by the Ethics Committee on the Care and Use of Laboratory Animals of Sun Yat-sen University (No. 00169218), and the experimental procedures were strictly performed in accordance with Legislation Regarding the Use and Care of Laboratory Animals of China. Mice were housed in a temperature controlled (20–25°C) and humidity-controlled (40–70%) barrier system with a 12-h: 12-h light-dark cycle.

After acclimatization for a week, one part of the mice (the control group, *n* = 8) were fed the common pellet diet while the other mice were fed high-fat diet for 2 weeks and then administrated with 50 mg/kg/d STZ by intraperitoneal injection after overnight fasting for 5 consecutive days to induce diabetes. The high-fat diet for mice is composed of 15.0% lard, 20.0% sucrose, 0.2% bile salt, 1.2% cholesterol, 10.0% modified casein, 0.6% sodium bicarbonate, 0.4% talc powder, and 52.2% basic diet. Normal diet is composed of vitamins, amino acids and routine nutritional components. STZ was freshly dissolved in sodium citrate buffer (pH = 4.0), prepared on the ice and protected from light before its injection. Mice exhibited polydipsia and polyuria symptoms of diabetes after STZ injection. All of the diabetic mice were fed high-fat diet until the termination of the experiment. The FBG levels were measured using a One-Touch glucometer (Johnson and Johnson, United States) 2 weeks after STZ injection. Mice with FBG value higher than 11.1 mM were considered as diabetic mice. Then the diabetic mice were randomized to four treatment groups (*n* = 8 for each group) based on the weight and FBG levels as follows: diabetic model group, PA treatment group (low dose: 150 mg/kg), PA treatment group (high dose: 300 mg/kg) and Met treatment group (195 mg/kg). Pae or Met was mixed with vehicle (0.5% sodium carboxymethyl cellulose), and the mice in Pae and Met group were administrated by gavage. The same volume of vehicle was given to the mice in both normal control group and diabetic group by gavage. The mice were given the assigned treatments for 6 days every week at 9:30–10:30 AM for 8 weeks.

### Blood Sample and Liver Tissue Collections

At the end of the experiment, animals were fasted for 12 h, and then the mice were anesthetized by injected with 1% pentobarbital sodium (dissolved in physiological saline) in dose of 35 mg/kg. The blood samples were collected by drainage from the retroorbital venous plexus after the FBG detection. Serum was obtained by centrifugation at 3000 *g* for 15 min and then kept at -80°C until use. The isolated liver tissues were quickly weighed and cut into parts, some of them were dipped into 10% formalin and embedded in paraffins for Oil Red O staining. The rest were quickly frozen in liquid nitrogen and subsequently stored at -80°C for long preservation.

### Lipid Profile and GSP Level in Serum

The TC, TG, LDL-C, HDL-C, and glycosylated serum protein (GSP) levels were measured with commercial available kits from Nanjing Jiancheng Biology Engineering Institute (China).

### TC, TG, and Glycogen Levels in Liver

The frozen liver tissues were thawed, dried and weighed for TC, TG and glycogen measurements according to the protocols (Jiancheng; Nanjing, China). Briefly, the sample is weighed precisely and for every 1 g of the sample, 9 mL homogenate medium should be mixed and fully homogenized with a homogenizer on ice. The homogenate treated with centrifugation at 2500 rpm for 10 min would be separated and supernatant is extracted for further detection. For the glycogen detection, the samples were mixed with the alkaline solution in test tube at ratio of Sample weight (mg):Alkaline volume (μL) = 1:3, placed in boiling water bath for 20 min, and followed by a extraction step with mixed solvent (chloroform: methanol = 2:1, v/v). Mingled thoroughly for 18 h at room temperature, the liquid spontaneously separated into three parts: water phase, tissue fragment and lipid phase from top to bottom. The water phase was carefully collected for glycogen detection.

### H&E Staining

The obtained liver tissues were cut and made into paraffin-embedded slices (3–4 μm), and then stained with hematoxylin and eosin (H&E) using the standard protocol. These slices were dried in an oven at 60°C overnight followed by deparaffinization, rehydration, hematoxylin and eosin staining in turn, and were finally dehydrated and covered with neutral balsam.

We assessed and scored the histological slides with a system of scoring the features of non-alcoholic fatty liver disease (NA) called the NAFLD Activity Score (NAS) (Brunt et al., 2011; Santiago-Rolon et al., 2015). The scores of each component of the NAS were listed as follows: steatosis (0–3), lobular inflammation (0–3), ballooning (0–2). High values of the aggregate NAS mean severe pathological changes.

### Oil Red O Staining

Fresh liver tissue was fixed in 4% paraformaldehyde for more than 24 h, and then immersed in 15% and 30% sucrose solution for dehydration. Filter paper were used to sip up water on tissue surface and OCT embedding medium was dipped surrounding the tissue parts for later slice preparation. The embedded tissues were cut into 8–10 μm, and clean glass slides were put above tissues slices to obtain them, kept at -20°C for longer preservation. The frozen slices were air-dried for 10 min, fixed in paraformaldehyde for 15 min, and pre-incubated with oil red O reagent for 15 min successively. After permeabilization for 15 min, tissue slices were incubated with oil red O again at 37°C for 2 h. Then they were differentiated with 75% alcohol for 2 s and counterstained with hematoxylin for 2 min. After washed by pure water, they were differentiated with 1% acid alcohol for several seconds and treated with ammonium hydroxide, and finally sealed with gelatin glycerin.

We assessed and scored the histological slides with NAS. The scores of each component of the NAS were listed as follows: steatosis (0–3), lobular inflammation (0–3), ballooning (0–2). High values of the aggregate NAS mean severe pathological changes.

### Cellular Oil Red O Staining

At first, Oil Red O was diluted in isopropanol at the concentration of 0.5%, and then mixed with ultrapure water by 3:2, and filtered for two times before use. Having been washed with PBS for two times, HepG2 cells were then fixed in paraformaledehyde for 10 min, stained with prepared Oil Red O solution for another 30 min, and finally observed by hematoxylin staining.

### MTT Assay

The 3-(4, 5-dimethylthiazol-2-yl)-2, 5-diphenyl tetrazolium bromide (MTT, Sigma, United States) was used to evaluate the effects of 10, 20, 40, 80, and 100 μM Pae on proliferation of HepG2 cells for 24 h. At the end of Pae treatment for 24 h, 0.5 mg/mL MTT was added for another 4 h of incubation. Then the culture medium was carefully removed and 200 μL DMSO which solubilizes the formed formazan crystals was added to each well. The OD value was detected by using a microplate reader (Bio-Tek, United States) at 570 nm.

### Cell Culture and Treatment

HepG2 cells (American Type Culture Collection, Rockville, MD, United States) were grown in high-glucose DMEM (Gibco, Invitrogen, United States) with 25 mM glucose and 10% (v/v) fetal bovine serum (Bovogen, United States) in a humidified incubator with 5% CO_2_. After overnight serum-deprivation, HepG2 cells were divided into different groups for different treatments. Preparation of palmatic acid (PA) solution: Sodium palmitate (Sigma, United States) was dissolved in ultrapure water at 70°C, and then mixed with 20% (w/v) BSA (low free fatty acid, Solarbio, China), the mixture were kept in water bath at 55°C for 1 h, then filtered after cooled to be the stock solution of 10 mM PA/20% BSA and stored at -20°C (Karaskov et al., 2006). The 10 mM PA/20% BSA solution was kept in water bath 55°C for 15 min before its use, and then diluted in DMEM to the concentration of 0.25 mM. Pae was dissolved in dimethyl sulfoxide (DMSO, Sigma, United States) as the mother liquor at 100 mM and diluted in DMEM to the corresponding concentrations. In the whole procedures of cell experiments, the final concentration of DMSO was kept lower than 1‰.

HepG2 cells were pre-treated with Pae at 10, 20, and 40 μM for 2 h, and followed by treating with 0.25 mM PA for 24 h. MK-2206 (Akt inhibitor, Selleck, United States) and SC79 (Akt activator, MCE, United States) were pre-treated for 1 h. The final concentration of BSA was 0.5%. Normal control cells were added 0.5% BSA, considering that PA was dissolved in BSA. The cells were collected after 10 min insulin (100 nM, Beyotime, Haimen, Jiangsu, China) stimulation for western blotting.

### Western Blotting

The lysis buffer was prepared with RIPA lysis buffer mixed with protease and phosphatase inhibitor cocktail. After various treatments, the lysis buffer was added to the plate to harvest cells after PBS washing for three times, whereas the tissue fragments were homogenized by a homogenizer in the lysis buffer. Then the tested cell and tissue samples were collected from the supernatant by centrifuging them at 1,2000 *g* for 15 min. Equal amount of protein samples were subjected to SDS-PAGE after BCA protein assay and transferred to PVDF membranes. After locked with 5% non-fat milk at room temperature for 1 h, the membranes were incubated with primary antibodies at 4°C overnight. Next day, these membranes were incubated for 2 h at room temperature with the corresponding secondary antibodies. Then the protein brands were visualized with enhanced chemiluminescence reagents by using GE ImageQuant LAS4000mini (Waukesha, WI, United States).

### Glucose Uptake

After various treatments for 24 h, the complete medium where HepG2 cells grew were replaced with serum-free medium for another 3 h. Fifty μM 2NBDG (Invitrogen, United States) and 1 μM insulin (Beyotime, China) were added for additional 1 h. The cells were harvested with trypsin digestion for centrifugation and then suspended in PBS buffer for flow cytometry detection at 488 nm.

### Small-Interfering RNA (siRNA)

Specific siRNA targeting Akt2 and negative control were synthesized by GenePharma (Shanghai, China). The sequences of efficient Akt2-siRNA which had been proved by our previous study (Hao et al., 2014) were listed as follows: sense: 5′-GCUCCUUCAUUGGGUACAATT-3′, antisense: 5′-UUGUACCCAAUGAAGGAGCTT-3′. The Akt2-siRNA was transfected into HepG2 cells using RNAiMAX transfection reagent according to the manufacturer’s instructions and then co-incubated for 48 h to harvest.

### Molecular Docking

The Autodock 4.2 (Morris et al., 2009) was used to carry out to dock ligands into a protein’s binding site. Pae was docked into the active site of Akt2 to explore the Akt2-paeonol interactions. The crystal structure of Akt2 was obtained from PDB (PDB code: 1O6L).

### Data Analysis

All experiments were performed at least three times with similar results. The collected data were analyzed using Graphpad Prism 5.0 and exhibited as mean ± SEM. Unpaired Student’s *t*-test was used for comparison between two groups. For multiple comparisons, data were analyzed by one-way ANOVA with Bonferroni *post hoc* test multiple comparisons. *P* < 0.05 was considered statistically significant.

## Results

### Paeonol Ameliorated Glucose and Lipid Metabolism Disorders and Alleviated the Liver Injury in Diabetic Mice

**FIGURE 1:**
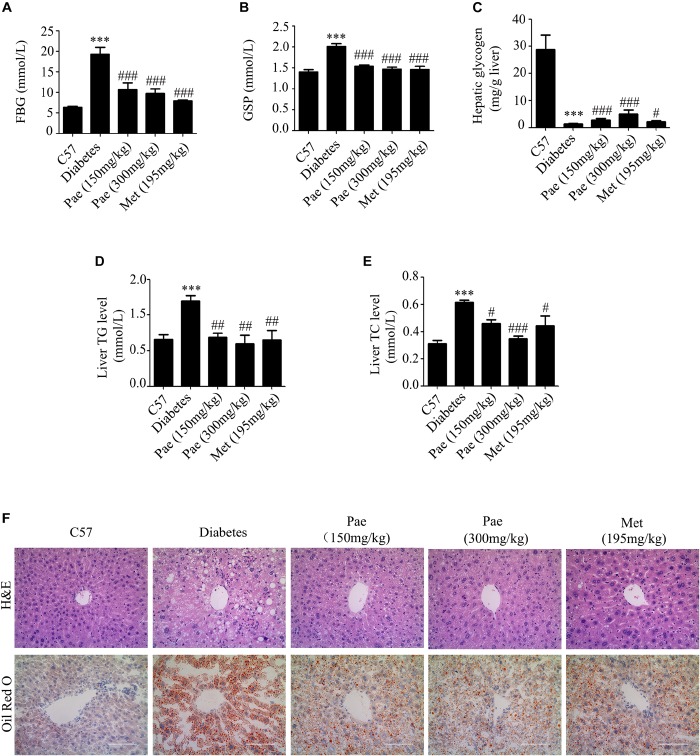
Paeonol ameliorated glucose and lipid metabolism and protected the liver in diabetic mice. **(A,B)** Measurements of FBG and GSP (*n* = 6-8). **(C–E)** Measurements of glycogen, TG and TC in diabetic livers (*n* = 6–8). **(F)** Histochemical analysis of liver tissues in diabetic mice, assessed by H&E and Oil Red O staining (×400 magnification). ^∗∗∗^*p* < 0.001 vs. C57; *^#^p* < 0.05, *^##^p* < 0.01, and *^###^p* < 0.001 vs. Diabetes.

In order to explore the effects of Pae on glucose and lipid metabolism in diabetic mice, we established diabetic mice model using high-fat diet with low-dose of streptozocin injection. Compared with the control group, diabetic mice presented elevated levels of serum TC, TG and LDL-C, which were all attenuated by Pae and Met treatments (Table 1). Pae significantly attenuated FBG and GSP levels (Figure 1A,B). Moreover, Pae increased glycogen content and decreased TG and TC levels in the liver (Figure 1C–E). H&E and Oil Red O staining both showed obvious pathological liver injury accompanied by the accumulation of fat and large distended lipid droplets in the livers of diabetic mice compared with C57 mice, which was remarkably reduced after Pae or Met treatment for 8 weeks (Figure 1F). C57 mice got low NAS (steatosis, lobular inflammation and ballooning all grade 0, NAS = 0), Diabetic mice got high NAS (steatosis grade 3; lobular inflammation grade 3; ballooning grade 2, NAS = 8), and diabetic mice with Pae or Met treatment got moderate NAS (steatosis, lobular inflammation and ballooning all grade 1, NAS = 3).

**Table 1:** The serum lipid levels of diabetic mice after PA treatment (*n* = 6–8).

Group	TG (mmol⋅L^-1^)	TC (mmol⋅L^-1^)	LDL-C (mmol⋅L^-1^)	HDL-C (mmol⋅L^-1^)
C57	0.80 ± 0.18	4.53 ± 0.42	0.5 ± 0.16	4.93 ± 0.49
Diabetes	2.01 ± 0.75^∗∗∗^	10.02 ± 1.60^∗∗∗^	1.64 ± 0.63^∗^	4.18 ± 0.50
Paeonol-low	0.71 ± 0.20^###^	7.47 ± 0.96^#^	0.74 ± 0.41^#^	3.91 ± 1.08
Paeonol-high	0.71 ± 0.11^###^	7.67 ± 1.02^#^	0.79 ± 0.37^#^	4.04 ± 0.59
Metformin	0.62 ± 0.16^###^	7.08 ± 1.40^##^	0.68 ± 0.49^##^	3.85 ± 1.12


*Data are represented as mean ± SD. TC, serum total cholesterol; TG, triglyceride; LDL-C, low-density lipoprotein cholesterol; HDL-C, high-density lipoprotein cholesterol. *^∗^p* < 0.05 and *^∗∗∗^p* < 0.001 vs. C57; *^#^p* < 0.05, *^##^p* < 0.01, and *^###^p* < 0.001 vs. Diabetes.*

**FIGURE 2:**
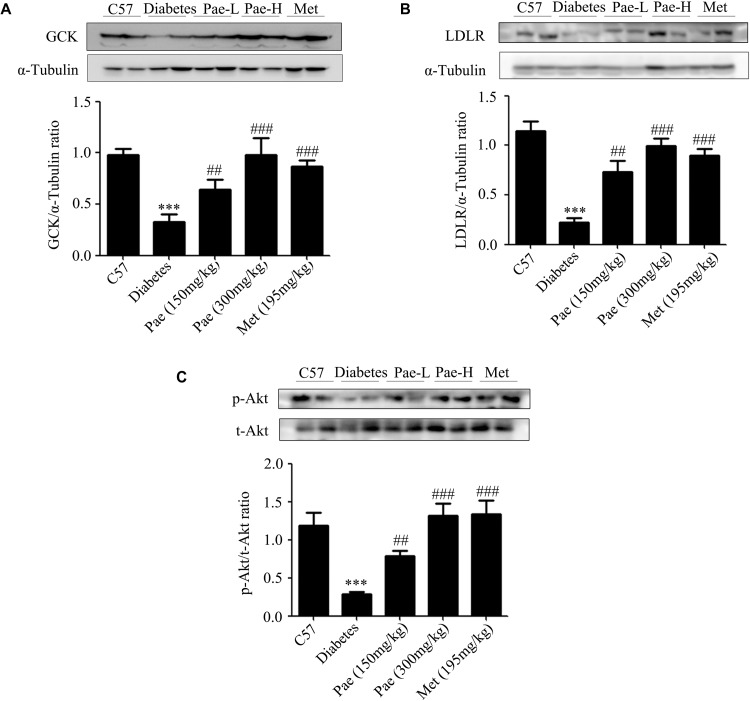
Paeonol upregulated the protein levels of GCK and LDLR and enhanced Akt phosphorylation in animals. **(A–C)** The protein expressions of GCK and LDLR and the phosphorylation level of Akt were measured by western blot analysis (*n* = 6–8). All experiments were performed at least three times with similar results. *^∗∗∗^p* < 0.001 vs. C57; *^##^p* < 0.01 and *^###^p* < 0.001 vs. Diabetes.

In the diabetic liver, the expressions of GCK and LDLR were downregulated, while these were increased by Pae treatment (Figure 2A,B). Considering Akt plays an important role in regulating glucose and lipid metabolism in diabetes, we detected the expressions of proteins relevant to glucose and lipid metabolism. As shown by Figure 2C, Pae increased the phosphorylation level of Akt.

The results *in vivo* indicated that Pae could improve the glucose and lipid metabolism in diabetic mice possibly by increasing the phosphorylation level of Akt and the protein levels of GCK and LDLR.

### Paeonol Improved Lipid Accumulation and Glucose Utilization in Insulin-Resistant HepG2 Cells

**FIGURE 3:**
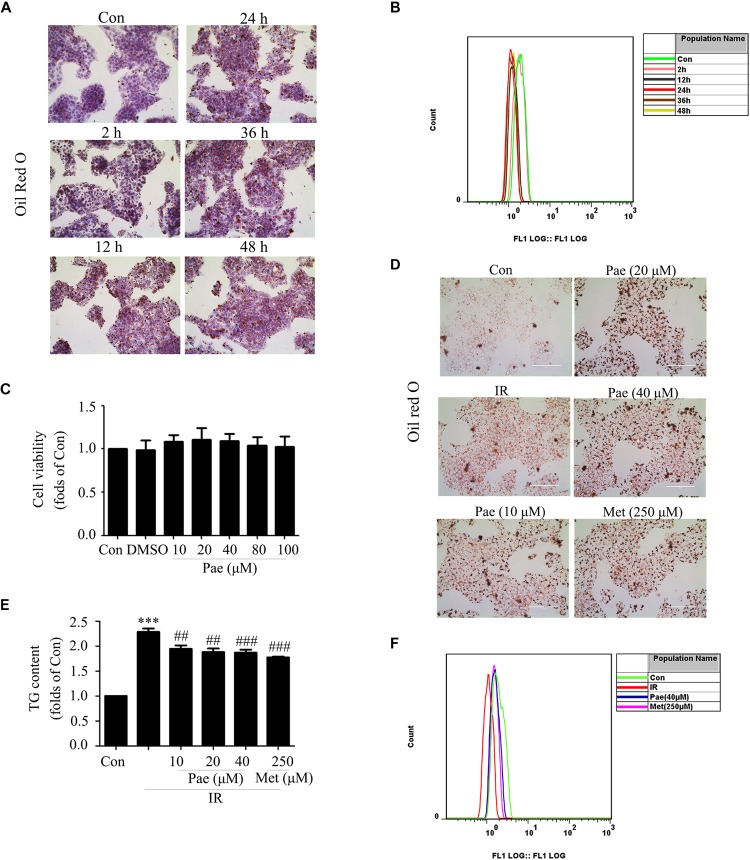
Paeonol improved lipid accumulation and glucose utilization in insulin-resistant HepG2 cells. **(A,B)** Oil Red O staining and glucose uptake of HepG2 cells treated by palmitic acid for different times. **(C)** Cell survival analysis through MTT assay of HepG2 cells treated with different concentrations of paeonol for 24 h. **(D)** The effects of paeonol on lipid accumulation assessed by Oil Red O. **(E)** Quantity analysis of triglyceride. **(F)** Glucose uptake assessment in paeonol-treated HepG2 cells under palmitic acid conditions. Con represents the control group; IR represents insulin-resistant group. ^∗∗∗^p < 0.001 vs. Con; ^##^p < 0.01 and ^###^p < 0.001 vs. IR.

To further confirm that Pae could attenuate glucose and lipid metabolism disorders and have an effect on protein kinase B, the insulin-resistant HepG2 cells model induced by PA was used. As reported by Hao et al. (2014), HepG2 cells were treated with 0.25 mM PA, and then, Oil Red O staining and glucose uptake experiments were used to verify whether the insulin resistant HepG2 cells were successfully constructed. As shown in Figure 3A,B, compared with control group, HepG2 cells treated with PA showed severe lipid accumulation and less glucose uptake at 12, 24, and 48 h. The glucose uptake reached the lowest level at 24 h. Taken together, HepG2 cells with 0.25 mM PA treatment for 24 h were employed in the following experiments.

In normal HepG2 cells, Pae showed no cytotoxicity below 100 μM for 24 h in the MTT assay (Figure 3C). Oil Red O staining showed that Pae treatment could reduce lipid accumulation at the concentrations of 10, 20, and 40 μM for 24 h (Figure 3D), and a quantity analysis of TG showed differences among treatment groups and the model group (Figure 3E). In insulin-resistant HepG2 cells, Pae improved glucose uptake at 40 μM, similar to 250 μM Met for 24 h (Figure 3F). These experiments confirm that Pae could attenuate glucose and lipid metabolism disorders *in vivo*.

### Paeonol Up-Regulated the Protein Expressions of GCK and LDLR as Well as the Phosphorylation Level of Akt in PA-Induced Insulin-Resistant HepG2 Cells

**FIGURE 4:**
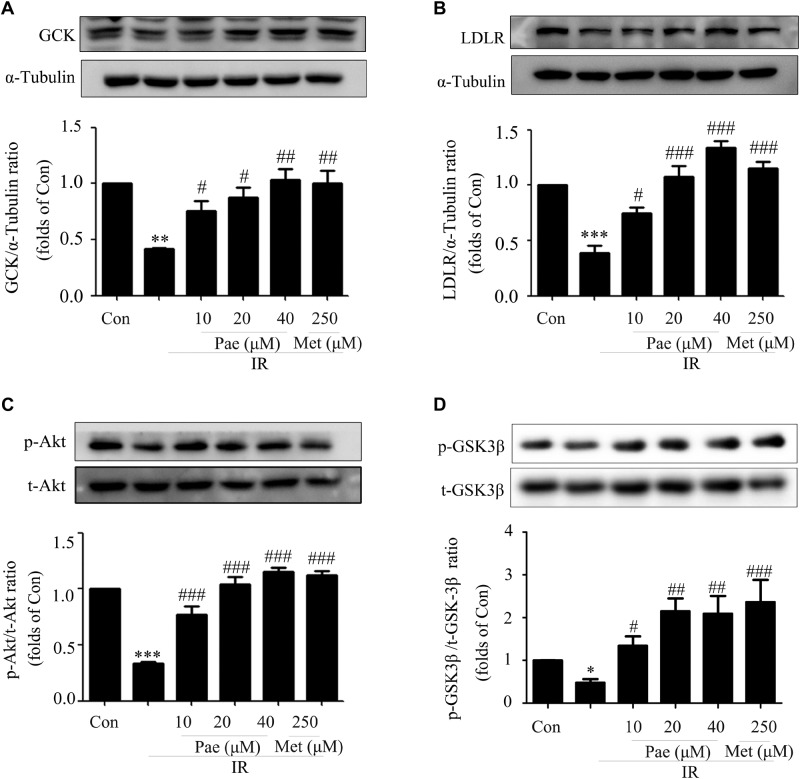
Paeonol regulated the phosphorylation of Akt and the protein contents of GCK and LDLR in insulin-resistant HepG2 cells. **(A,B)** GCK and LDLR expression levels induced by paeonol under IR. **(C)** Akt phosphorylation level in HepG2 cells. **(D)** p-GSK-3β content measured by western blot. All experiments were performed at least three times with similar results. *^∗^p* < 0.05, *^∗∗^p* < 0.01, and *^∗∗∗^p* < 0.001 vs. Con; *^#^p* < 0.01, *^##^p* < 0.01, and *^###^p* < 0.001 vs. IR.

In insulin-resistant HepG2 cells, Pae treatment for 24 h up-regulated the expressions of GCK and LDLR (Figure 4A,B). Moreover, the phosphorylation level of Akt and its downstream protein GSK-3β were also increased (Figure 4C,D). These were consistant with the *in vivo* results. Therefore, an assumption that Pae regulates GCK and LDLR levels by increasing Akt phophorylation arouses our interests.

### Paeonol Regulated GCK and LDLR Through Akt

To further verify the possible effects of Pae on GCK and LDLR through Akt, we carried out a series of experiments using Akt inhibitor MK2206 (10 μM) and Akt2-siRNA, because Akt2 is the major subtype of protein kinase B in liver. SC79 (5 μM), a specific Akt agonist, was used as the positive control.

**FIGURE 5:**
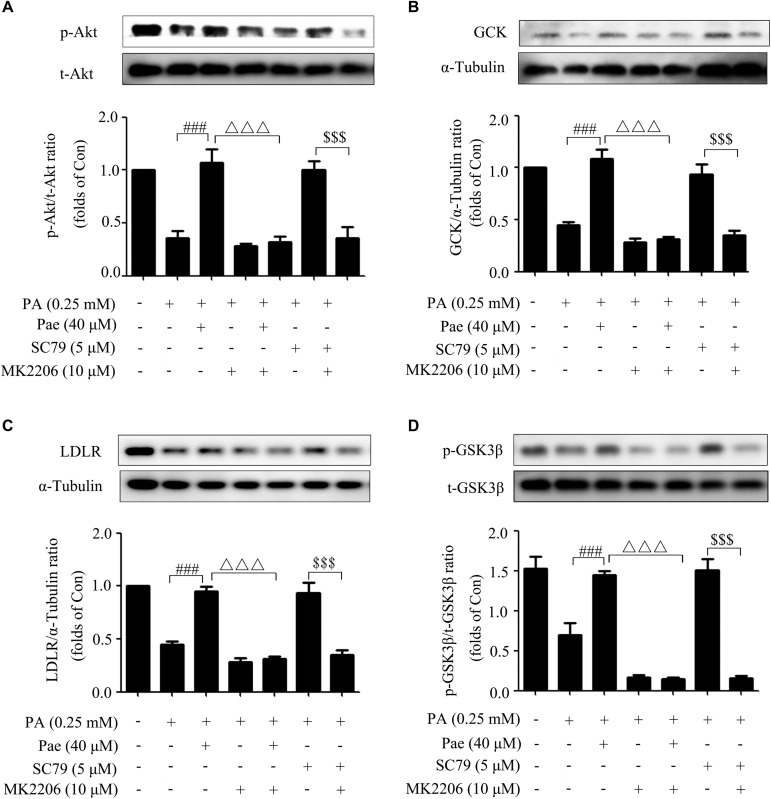
Paeonol regulated LDLR and GCK through Akt. **(A)** MK2206 blocked the paeonol-induced up-regulation of p-Akt. **(B,C)** MK2206 inhibited the elevated levels of GCK and LDLR induced by paeonol or SC79. **(D)** MK2206 blocked the paeonol-induced up-regulation of p-GSK-3β. All experiments were performed at least three times with similar results. *^###^p* < 0.001 vs. PA (0.25 mM); ^Δ Δ Δ^ *p* < 0.001 vs. PA + Pae; ^$$$^*p* < 0.001 vs. PA + SC79.

Concomitant with the remarkable inhibition of Akt phosphorylation, MK2206 canceled the increase of Akt phosphorylation induced by Pae or SC79 compared with the control group (Figure 5A). Meanwhile, after Akt was inhibited by MK2206, the up-regulated effects of Pae on GCK and LDLR expressions disappeared (Figure 5B,C). We also detected the phosphorylation level of GSK3β, which is a direct downstream target of Akt and regulates glucose metabolism, and found that p-GSK3β was increased after Pae or SC79 treatment under insulin-resistant states. However, this increase was diminished by MK2206 incubation (Figure 5D).

**FIGURE 6:**
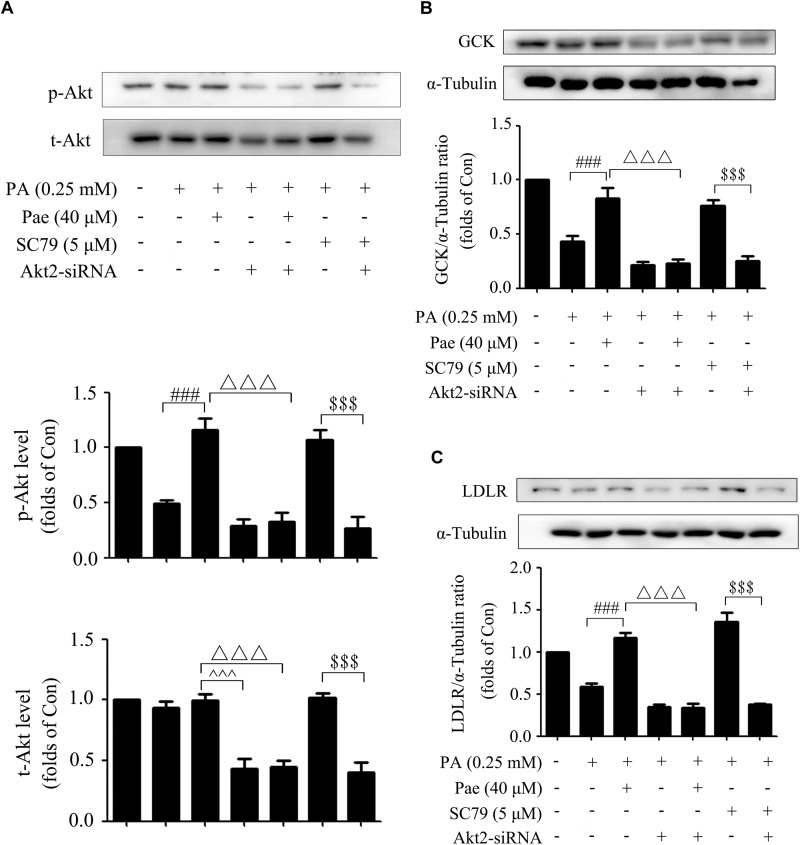
The effects of Akt2-siRNA on GCK, LDLR and p-Akt. **(A)** Paeonol-induced levels of t-Akt and p-Akt were assessed after treatment with Akt2-siRNA under insulin resistant conditions. **(B,C)** Akt2-siRNA abolished the effects of paeonol on GCK and LDLR. All experiments were performed at least three times with similar results. *^###^p* < 0.001 vs. PA (0.25 mM); ^△△△^*p* < 0.001 vs. PA + Pae; ^$$$^*p* < 0.001 vs. PA + SC79; ^∧∧∧^*p* < 0.001 vs. PA + Akt2-siRNA.

The silencing efficiency of Akt2-siRNA was confirmed in the previous study (Hao et al., 2014). As shown by Figure 6A, Akt2-siRNA caused a sharp decline in Akt expression as well as its phosphorylation level. Similarly, knockdown of Akt2 by siRNA abrogated the upregulated effects of Pae on GCK and LDLR expressions under insulin-resistant conditions (Figure 6B,C).

Accordingly, our *in vitro* study elucidated that Pae could improve glucose and lipid metabolism in PA-induced insulin-resistant HepG2 cells by upregulating the GCK and LDLR expressions through increasing the phosphorylation level of Akt.

### Molecular Docking

**FIGURE 7:**
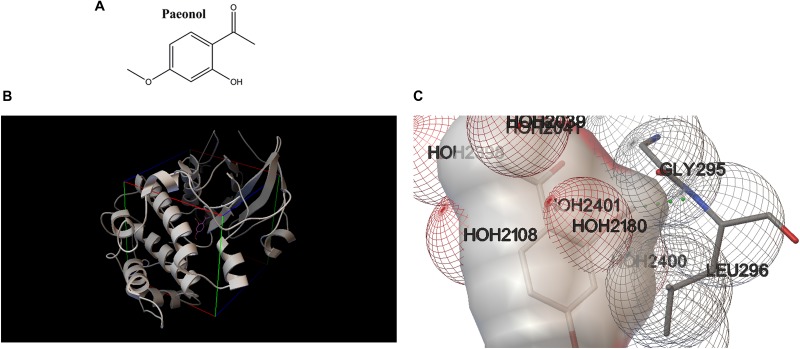
Molecular docking revealed a direct combination between paeonol and Akt2 crystal structure (PDB code: 1O6L). **(A)** Chemical structure of paeonol. **(B)** Docking pose of paeonol in the active pocket of the Akt2 crystal structure. **(C)** Paeonol formed one steady hydrogen bond with Akt2.

To better figure out the relationship between Pae and Akt, Pae was docked into the active pocket using Autodock 4.2. The molecular docking assay indicated that Pae (Figure 7A) could bind to the active pocket of the Akt2 crystal structure (PDB code: 1O6L) (Figure 7B), but formed only one steady hydrogen bond with Akt2 in Gly295 amino residue (Figure 7C), because the simple structure of Pae limits the number of hydrogen bond that can be formed. The interaction between Akt2 and Pae might impact the phosphorylation of Akt, and then improved glucose and lipid metabolism.

## Discussion

Most researches of Pae focus on its anti-inflammatory and anti-oxidative activities (Chou, 2003; Ping et al., 2014; Jin et al., 2016), and there are a few reports of its potential use for the treatment of diabetes (Lau et al., 2007; Juan et al., 2010). Pae was reported to prevent diabetes complications such as diabetic encephalopathy (Liu et al., 2013). Recently, Zhang et al. (2018) reported that Pae could delayed the progression of diabetic nephropathy. However, the hypoglycemic activity of Pae is rarely elucidated and the underlying mechanisms of its anti-diabetic activity remain largely unclear. In the present study, we extended these previous investigations and provided evidence that Pae not only had the hypoglycemic effects but also showed hypolipidemic effects for the first time in diabetic mice model and insulin-resistant HepG2 cells. *In vivo*, mice were fed with high-fat diet to induce IR (Ribeiro et al., 2012). In the meanwhile, STZ was used to selectively impair the pancreatic cells, causing insulin secretion deficiency with increased level of blood glucose (Huang et al., 2010; Dong and Chen, 2013). The observed increments of FBG, GSP, TC, TG, and LDL-C plus with lipid accumulation in livers proved the success of high-glucose and lipid-diabetic model. However, they were all ameliorated after treatment of Pae for 8 weeks, indicating the improving effect of Pae on glucose and lipid disorders. The hypoglycemic effect of Pae was consistant with the reported results about the improving oral glucose tolerance of Pae (Lau et al., 2007). *In vitro*, the Pae treatment group showed increased glucose utilization in insulin-resistant HepG2 cells, consistent with the previous study (Hsu et al., 1997; Lau et al., 2007). Meanwhile, Pae ameliorated the severe lipid accumulation in insulin-resistant HepG2 cells. On the whole, we confirmed that Pae could ameliorate glucose metabolism in diabetic models. More importantly, our research also found that Pae exhibited the ameliorating role in regulating lipid metabolism *in vivo* and *in vitro*.

After we confirmed that Pae could ameliorate glucose and lipid metabolism, the underlying mechanisms of its effects needed to be explored. According to our previous study, we found LDLR and GCK are significantly increased by polydatin in STZ-induced diabetic rats (Hao et al., 2014). As reported, LDLR is a key receptor for maintaining cholesterol homeostasis in mammals. Decreased LDLR is recognized as the pivotal factor responsible for hypercholesterolemia (Go and Mani, 2012), and LDLR^-/-^ mice shows obvious changes in hepatic lipid contents (Kockx et al., 2016). Besides, hepatic GCK catalyzes the phosphorylation of glucose to glucose to 6-phosphate (G6P), a step which is essential for glycolysis, glycogen synthesis and lipogenesis (Dentin et al., 2004). GCK is shown to decrease in diabetic mice with IR (Xie et al., 2011). Therefore, we detected the protein levels of LDLR and GCK in diabetic livers. Our data indeed indicated that LDLR and GCK were increased by Pae in STZ-induced diabetic mice, which were concordant with the reported results about polydatin ameliorating glucose and lipid disorders through up-regulating GCK and LDLR levels (Hao et al., 2014; Wang et al., 2016). On the other hand, Akt is considered as a key regulator relevant to glucose metabolism in the insulin signaling cascade (Whiteman et al., 2002). Akt regulates lipogenesis through parallel mTORC1-dependent and independent pathways (Yecies et al., 2011). As expected, we found that Pae treatment increased the phosphorylation level of Akt. Our results showed that Pae improved glucose and lipid metabolism by increasing the phosphorylation level of Akt and expressions of GCK and LDLR.

While how Pae regulated the expressions of LDLR and GCK was unclear. Several studies have indicated that Akt participates in the regulation of lipid metabolism by LDLR through SREBP-1c activation (Horton et al., 2002; Guo et al., 2011), and our study showed that Akt indeed influenced SREBP-1c expression (see Supplementary Figure S1). Accumulating data also manifest that GCK (Hagiwara et al., 2012; Matveyenko et al., 2012; Song et al., 2017) acts as the intermediate of Akt in regulating glucose metabolism. Therefore, Akt aroused our interests and we made a reasonable speculation that Pae might increase LDLR and GCK expressions through the activation of Akt. This would improve glucose and lipid metabolism as well as IR, which acts as hypoglycemic and lipid-lowering roles in diabetic mice and insulin-resistant HepG2 cells. *In vitro* experiments were designed to demonstrate our inference.

*In vitro*, we first established an insulin-resistant cell model induced by PA (Karaskov et al., 2006; Hao et al., 2014). As expected, Pae not only up-regulated protein levels of GCK and LDLR but also increased the phosphorylation level of Akt in insulin-resistant cells, which was consistent with animal experiments. What’s more, we detected the phosphorylation level of GSK3β, a direct downstream protein of Akt, and found that its phosphorylation level was increased by Pae treatment compared with PA treatment group. These results indicated that Pae may increase GCK and LDLR levels by activating Akt. Akt inhibition was performed to verify the above hypothesis. The up-regulating effects of Pae on LDLR and GCK disappeared in the PA + MK2206 group. In the experiment of Akt knockdown, the three human Akt isoforms (Akt1, 2, and 3) differ only in a few amino acids (Okano et al., 2000; Gonzalez and McGraw, 2009). Akt1 is reported to be widely expressed in the whole body, Akt2 expression markedly increased in insulin-dependent tissues and Akt3 mostly observed in brain (Chen et al., 2001). Previous studies reveal that Akt2 knockdown results in a syndrome similar to diabetes (Cho et al., 2001b) and Akt2 is indispensable for glucose homeostasis (Cho et al., 2001a). Thus, we chose Akt2 small interfering RNA for further verification. Similarly, Akt2-siRNA treatment caused a sharp decline in Akt expression as well as its phosphorylation level. After Akt2 was interfered by siRNA, the Pae -induced up-regulation of LDLR and GCK under IR conditions disappeared. These results demonstrated that Pae was involved in the regulation of LDLR and GCK by activating Akt.

Most importantly, from the results of the present study, we identified, for the first time, Pae regulated glucose and lipid metabolism through activating Akt *in vivo* and *in vitro*, and within these models Pae induced a variety of metabolic effects consistent with Akt activation. These included increased phosphorylation levels of Akt and GSK3β; increased expression of LDLR involved in lipid metabolism; and increased expression of GCK involved in glucose metabolism.

It was intriguing to discuss the two positive controls, Met and SC79. Met is used in clinical treatment for type 2 diabetes as a first-line drug with remarkable effects (Palmer and Strippoli, 2018), that was the reason why we choose it as the positive drug in animal experiments. Based on the data presented here, Met could attenuate glucose and lipid metabolism in diabetic mouse models and insulin-resistant HepG2 cells, similar to previous study that Met improves insulin sensitivity and causes lipid lowering effects in animal models of IR (Saenz et al., 2005). In our study, Met was showed to increase the phosphorylation level of Akt, consistent with previous study (Zheng et al., 2015; Xu et al., 2016). These data showed that the choice of choosing Met as a positive drug was appropriate for proving hypoglycemic and hypolipidemic effects of Pae in diabetic and insulin-resistant models. However, in the experiments of Akt inhibition and knockdown, SC79, a specific Akt activator (Zhang et al., 2016; Liu et al., 2018), would be better as the positive control. All in all, two positive drugs reflected the fact that Pae regulated GCK and LDLR through its effects on Akt. What’s more, according to the molecular docking assay, one steady hydrogen bond was observed to be formed between Pae and active pocket of the Akt2 crystal structure, because the simple structure of Pae limited the number of hydrogen bond that can be formed, these results indicated that Pae might bind to Akt2 and change the conformation of Akt2, thereby inducing its downstream signal transduction.

## Conclusion

In sum, Pae not only had the hypoglycemic effects but also showed hypolipidemic effects and its underlying mechanisms were closely associated with Akt in insulin-resistant models. This study provided preliminary experimental evidence for the therapeutical potentials of Pae in the intervention of IR and regulation of glucose and lipid metabolism disorders.

## Data Availability

The datasets used and/or analyzed during the current study are available from the corresponding author on reasonable request.

## Ethics Statement

All experimental procedures were carried out in accordance with the China Animal Welfare Legislation, and approved by the Ethics Committee on the Care and Use of Laboratory Animals of Sun Yat-sen University.

## Author Contributions

FX and HH designed and performed the experiments, acquired and analyzed the data, and drafted the manuscript. HX and YY helped to perform the animal experiments and prepared the manuscript. MZ and LC helped to perform the *in vitro* experiments and computer-assisted software. ZC has been involved in drafting the manuscript and revising it critically for important intellectual content. RL participated in drafting the revised manuscript and helped to conduct the additional experiments. PL contributed to reagents, materials, and analysis tools. All authors read and approved the final manuscript.

## Conflict of Interest Statement

The authors declare that the research was conducted in the absence of any commercial or financial relationships that could be construed as a potential conflict of interest.

## Abbreviations

Akt: Serine/threonine kinase B
BSA: bovine serum albumin
DMEM: Dulbecco’s modified Eagle’s medium
FBG: fasting blood glucose
GCK: glucokinase
GSK-3β: glycogen synthase kinase-3 beta
HDL-C: high density lipoprotein cholesterol
LDL-C: lower density lipoprotein cholesterol
LDLR: low-density lipoprotein receptor
Met: metformin
MTT: 3-(4, 5-dimethylthiazol-2-yl)-2, 5-diphenyl tetrazolium bro-mide
PA: palmitic acid
Pae: paeonol
TC: total cholesterol
TG: triglyceride
