# Supplementary figures and images for: Paeonol Ameliorates Glucose and Lipid Metabolism in Experimental Diabetes by Activating Akt
Source: Front Pharmacol. 2019 Mar 19;10:261. doi: 10.3389/fphar.2019.00261 (PMC6433795; doi:10.3389/fphar.2019.00261)

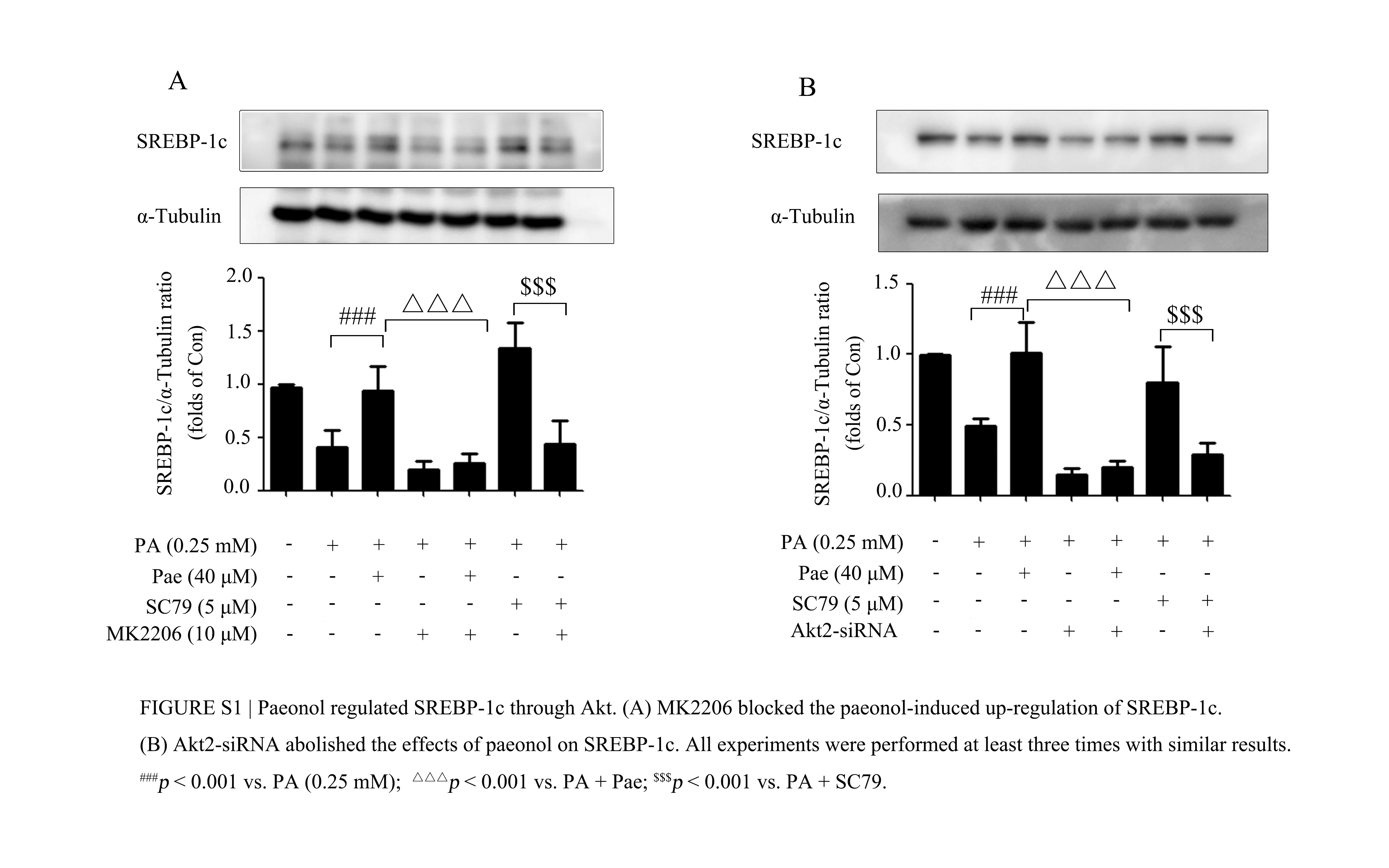

Supplement: Supplementary file 1 [file Image_1.tif]
